# A qualitative examination of naloxone access in three states: Connecticut, Kentucky, and Wisconsin

**DOI:** 10.1186/s12889-022-13741-5

**Published:** 2022-07-19

**Authors:** Antoinette L. Spector, Carol L. Galletly, Erika A. Christenson, H. Danielle Green Montaque, Julia Dickson-Gomez

**Affiliations:** 1 Department of Rehabilitation Sciences and Technology, College of Health Sciences, University of Wisconsin-Milwaukee, P.O. Box 413, Milwaukee, WI 53201 USA; 2 Department of Psychiatry and Behavioral Medicine, Center for AIDS Intervention Research, Medical College of Wisconsin, 2701 N. Summit Ave, Milwaukee, WI 53202 USA; 3 Center of Excellence in Women’s Health, Boston, Medical Center/BUSM, 801 Massachusetts Avenue, Boston, MA 02118 USA; 4 Institute for Community Research, 2 Hartford Square West, 146 Wyllys St., Suite 100, Hartford, CT 06106 USA; 5 Division of Epidemiology, Institute for Health and Equity, Medical College of Wisconsin, 8701 W. Watertown Plank Rd, Milwaukee, WI 53226 USA

**Keywords:** Naloxone, Narcan®, Access, Opioid overdose prevention, Qualitative research

## Abstract

**Background:**

Prevention of opioid-involved overdose deaths remains a public health priority in the United States. While expanding access to naloxone is a national public health strategy, it is largely implemented at the state and local level, where significant variability in policies, resources, and norms exist. The aims of the current study were to examine the social context of naloxone access in three different states (Connecticut, Kentucky, Wisconsin) from the perspectives of key informants (first responders, harm reduction personnel, and pharmacists), who play some role in dispensing or administering naloxone within their communities.

**Methods:**

Interviews were conducted with key informants who were in different local areas (urban, suburban, rural) across Connecticut, Kentucky, and Wisconsin. Interview guides explored the key informants’ experiences with administering or dispensing naloxone, and their perspectives on opioid overdose prevention efforts in their areas. Data analysis was conducted using multistage inductive coding and comparative methods to identify dominant themes within the data.

**Results:**

Key informants in each of the three states noted progress toward expanding naloxone access, especially among people who use opioids, but also described inequities. The key role of harm reduction programs in distributing naloxone within their communities was also highlighted by participants, as well as barriers to increasing naloxone access through pharmacies. Although there was general consensus regarding the effectiveness of expanding naloxone access to prevent overdose deaths, the results indicate that communities are still grappling with stigma associated with drug use and a harm reduction approach.

**Conclusion:**

Findings suggest that public health interventions that target naloxone distribution through harm reduction programs can enhance access within local communities. Strategies that address stigmatizing attitudes toward people who use drugs and harm reduction may also facilitate naloxone expansion efforts, overall, as well as policies that improve the affordability and awareness of naloxone through the pharmacy.

**Supplementary Information:**

The online version contains supplementary material available at 10.1186/s12889-022-13741-5.

## Background

Opioid-involved overdose deaths have been on the rise over the last two decades and have contributed to drug overdose deaths becoming one of the leading causes of injury-related mortality in the United States (U.S.) [1, 2]. In 2018, nearly 70% of all drug overdose deaths in the U.S. involved an opioid and over two million people were living with an opioid use disorder [3, 4]. In response to the opioid crisis, the U.S. has prioritized expanded access to naloxone [5]—also known by the commercial name, Narcan®—an opioid antagonist medication that can reverse the effects of an overdose and has no abuse potential [6]. Naloxone was initially approved for use in hospital and emergency response settings, but a take-home version of naloxone is a newer application meant to prevent opioid-involved overdoses outside of the healthcare environment [7]. The U.S. Department of Health and Human Services has recommended expanded community access to naloxone as one of the top strategies for addressing the opioid epidemic [8]. Efforts to increase naloxone utilization have involved distribution programs at syringe services programs (SSPs) and public health departments, access through pharmacies without needing a prescription, and administration of naloxone by first responders.

SSPs have been ramping up their distribution of naloxone since the mid-1990s and are the most common mode for community members, particularly people who use drugs (PWUD), to obtain naloxone without a prescription [9–11]. SSPs can target individuals who may be at increased risk for overdose, such as people who inject drugs (PWID), as well as community members who may be bystanders to an overdose event [6]. Naloxone distribution through harm reduction organizations, like SSPs, has been shown to be a safe and cost-effective option to reduce opioid-involved overdose fatality rates at the local level [12–14].

While SSPs have been the primary method to distribute naloxone to PWUD, access can vary between and within states. In 2018, there were reported to be over 300 SSPs operating in the U.S. and the District of Columbia, but southern, midwestern, and predominantly rural states were noted to have the least availability [15, 16]. Between 2014 and 2019, the majority of states took actions to eliminate any ambiguity regarding the legality of services provided by SSPs and, notably, many of these legal changes also happened concurrently with states expanding access to naloxone [16]. However, greater legal clarity did not necessarily coincide with states allocating additional funding to support SSPs (who are unable to receive funding at the federal level) or an increase in community support at the local level [16–18]. Moreover, since naloxone is considered a prescription medication in most states, and thus requires a physician to authorize its distribution, it can be challenging for harm reduction organizations to gain medical authorization for naloxone distribution [19].

Policies have been implemented in every U.S. state and the District of Columbia to improve naloxone access through pharmacies and are in alignment with the advisory issued by the U.S. Surgeon General in 2018 promoting the possession of naloxone [20]. Prior to these changes, for naloxone to be obtained in the pharmacy, an order from a qualified prescriber was required. The two most common mechanisms to improve naloxone access through the pharmacy have been to authorize pharmacists to prescribe naloxone, or to implement a standing physician order [21]. New Mexico was among the first states to have a naloxone access law that grants pharmacists prescriptive authority, or the ability to dispense naloxone without a prescription from a physician [22, 23]. When pharmacists are given prescriptive authority, some states may require that they obtain a specialized certification, while others allow unrestricted authority to dispense naloxone [24]. In the case of a standing order, the primary method is for the state medical director to issue an order that allows designated pharmacists to dispense naloxone [25].

Previous research has shown that expanded naloxone access laws are an effective way to increase consumer access to naloxone in retail pharmacies and to reduce opioid-involved overdose deaths, particularly when granting direct authority to pharmacists [26–30]. Yet, there are several barriers to pharmacy-based naloxone distribution, including pharmacists’ negative attitudes toward PWUD, a lack of clarity regarding their role in opioid overdose prevention, how pharmacies and pharmacists’ workload are structured, variability in insurance coverage for naloxone, and having a certified pharmacist onsite within states who have this requirement [22, 31, 32]. On the other hand, the accessibility of pharmacies can be an advantage to naloxone access expansion, especially in the absence of local harm reduction organizations, such as in rural communities [22]. Moreover, there is emerging evidence that pharmacy-based harm reduction interventions are a feasible approach to opioid overdose prevention [33].

Communities have increasingly expanded naloxone access to non-paramedic first responders, such as police officers, firefighters, and emergency medical technicians [34, 35]. Non-paramedic first responders are often the first to arrive on the scene of an overdose event but were historically not permitted to administer medications [34, 36]. When non-paramedic first responders had to wait for paramedics to arrive to administer naloxone, it could be problematic for non-metropolitan communities who had fewer emergency medical resources [34, 36, 37].

Like pharmacists, many state offices that oversee emergency medical services (EMS) have changed their protocols to allow local medical directors to issue standing orders for non-paramedic first responders (i.e., law enforcement, firefighters, emergency medical technicians) to administer naloxone [34]. Fire and police departments have partnered with health departments to receive training in naloxone administration, as well as increasing their capacity to equip firefighters and police officers with naloxone [34]. There is emerging evidence that expanded naloxone access among first responders has been associated with fewer opioid-related overdose deaths, and has been bolstered by the implementation of Good Samaritan laws, which provide legal protection to individuals who contact EMS to respond to an overdose event [27, 38]. Yet, ambivalence regarding the role of law enforcement in responding to overdoses, negative attitudes of first responders toward PWUD, and mistrust of first responders, on the part of PWUD, can present barriers to naloxone access expansion at the local level [37, 39].

As opioid overdose prevention strategies continue to evolve across communities in the U.S., the current study’s purpose was to explore the shifting landscape of naloxone access from the perspectives of key informants (KIs), who each play some role in distributing or administering naloxone: first responders (paramedic and non-paramedic), harm reduction personnel (SSPs and public health agencies), and pharmacists. The study uses qualitative interviews with these KIs, who were in either Connecticut (CT), Kentucky (KY), or Wisconsin (WI)—states that each have differing approaches to naloxone expansion. It explores differences by sector, and between states and local areas, regarding changes in community access to naloxone and the social context of naloxone expansion efforts, including the perceived consequences of these strategies. Research questions to be answered include the following: How has naloxone access changed within each state? How have any changes in naloxone access differed locally? What are KI’s perspectives on the consequences (intended and unintended) of naloxone expansion efforts?

## Methods

### Overview

The current study is part of a larger project funded by the National Institute on Drug Abuse, Project LEAD (Laws to Eliminate Abuse and Diversion). Project LEAD is a three-state study (CT, KY, WI) that examined and compared factors that influence the relationship between opioid-related laws and policies, and the transition from prescription opioids to heroin, fentanyl, and injection drug use. In particular, the study aimed to examine differing state laws and policies regarding harm reduction activities, such as those governing syringe services programs and pharmacy distribution of naloxone. In addition, an urban, suburban, and rural area were selected in each state to examine the role of the local context on the transition from prescription opioids to heroin, fentanyl, and injection drug use. These geographic areas were selected based on high rates of opioid prescription and/or overdose deaths and that represent urban, suburban/smaller urban, or rural areas. Population size and distance to a metropolitan center were used to differentiate local areas, with suburban areas reflecting a small city less than 20 miles to an urban center in each state. Rural areas reflect counties in each state that were at least 20 miles from the selected urban area. The rural area in CT was approximately 20 miles from the selected urban area; KY and WI were approximately 120 miles from the selected urban areas. The Project LEAD methods have been described in detail elsewhere [40].

### Current study

The current study used data from KIs who played some role in either administering or distributing naloxone in the community and included first responders, harm reduction personnel, and pharmacists. The primary role of the study’s KIs was to help determine factors, including state laws and local context, that have influenced opioid use behaviors in their communities. Prior research has shown that KIs have can provide an accurate assessment of the drug use context in their communities and have been used extensively in epidemiological and ethnographic studies for this purpose [41].

The expertise of the research teams in each state was used to identify an initial list of KIs. To be eligible, KIs had to be at least 18 years old and currently working in the specific sectors of interest. Purposive sampling was done in each local area within each state to ensure adequate coverage of each sector. Participant referral was used to recruit additional KIs. Potential participants were contacted using an email message, which provided a brief description of the study and explained why they were being asked to participate. If they expressed interest, interviewers scheduled a time to conduct either a face-to-face or phone interview. All participants were told that their participation was voluntary and would be kept confidential. Each participant provided written informed consent to participate in the study. Interviews lasted approximately 30 to 60 min and were audio recorded. All procedures were approved by the Institutional Review Board at the Medical College of Wisconsin.

### Interview content

Although the interview guides and probes varied by sector, each KI in the current study was specifically asked about their specific role in administering or distributing naloxone, and their overall views regarding their community’s response to opioid overdose prevention. Detailed information on specific interview questions and probes is available in Additional File 1. First responders were asked about the frequency of opioid overdose events, the availability of naloxone within the community, and if they had observed any changes in these areas. They were also asked if they were able to dispense naloxone and whether they did any community outreach involving opioid overdose prevention. Harm reduction personnel were similarly asked about opioid overdoses and if they had observed any changes in the frequency of these events. They were asked about the types of harm reduction services they provided, including whether their organization distributed naloxone and provided opioid overdose prevention education, and their perceptions regarding the overall effectiveness of their harm reduction interventions. Harm reduction personnel were asked if there were any gaps in current overdose prevention strategies within their communities. Pharmacists were asked if they dispensed naloxone and the rationale behind their capability to do so. They were asked about any other opioid overdose prevention efforts that occurred in their community.

### Data analysis

All interviews were transcribed verbatim. A collaborative, multi-stage approach for data analysis was used to develop a codebook [42, 43]. First, the multi-state research team selected an initial transcript, which was used to develop a preliminary list of inductive codes identified from the interview. The preliminary coding list was then applied to three additional transcripts—which were purposively selected to reflect different KI experiences (e.g., state, local area, sector)— and refined until the multi-state research team reached consensus on a final list of codes, their meanings, and the procedures for assigning them to text data [42, 44]. MAXQDA software was used to apply the final list of codes to all the transcripts, which was completed by six members of the multi-state research team. The coding, development of new codes, and analytic memoing (or notes reflecting how team members were interpreting the data) were tracked by the six-person team to identify and clarify the explicit meanings of the data, as well as to capture underlying patterns and assumptions [44]. Bi-weekly team meetings were held for troubleshooting and quality checks and included the principal investigator of the study.

To address the current study’s aims, constant comparative methods were used to explore participants’ experiences distributing or administering naloxone [42]. First, the analysis examined what KIs had to say regarding changes in naloxone access within their communities and compared their experiences by sector (first responder, harm reduction, pharmacist) and the different states and areas in which they were located. The analysis then explored what KIs attributed to any changes in naloxone access and their perspectives on the social context of naloxone access expansion within their communities.

## Results

The sample consisted of 27 first responders, 16 harm reduction personnel, and 9 pharmacists in CT, KY, and WI for a total of 52 KIs. Variation in subsample size reflect differences in recruitment by state (i.e., research team contacts and participant referral) and the cessation of KI recruitment when the study team agreed that saturation had been achieved.

Table 1 summarizes the participants’ characteristics overall and by state. There was a nearly even distribution of KIs across states with 31% from both CT and KY and 38% from WI. About half of the KIs worked in an urban area (52%) followed by 29% and 19% being located in suburban and rural areas, respectively.

**Table 1:** Characteristics of key informants

	n (%)	Connecticut	Kentucky	Wisconsin
Overall	52 (100)	16 (31%)	16 (31%)	20 (38%)
Geographic area				
Rural	10 (19)	3	3	4
Suburban	15 (29)	3	5	7
Urban	27 (52)	10	8	9
Key informant category				
First responder	27 (52)	6	11	10
Harm reduction	16 (31)	8	3	5
Pharmacist	9 (17)	2	2	5

The analyses explored changes in the local context of naloxone access in CT, KY, and WI from the perspectives of KIs who either distributed or administered naloxone within their communities. The results are organized around five major themes that were identified in the data and are summarized in Table 2. The initial theme centers around changes in naloxone across the three states and local areas. While KIs generally indicated progress in getting naloxone into the hands of more individuals, particularly PWUD, they also expressed concerns regarding inequities in access. The next theme sheds light on the central role of harm reduction organizations in promoting community access to naloxone. Conversely, the fourth theme highlights the challenges to expanding naloxone access through the pharmacy. The final theme focuses on how KIs grappled with stigmatizing attitudes toward PWUD and a harm reduction approach to overdose prevention, and their related concerns regarding the unintended consequences of naloxone expansion efforts. Excerpts from interviews are used to illustrate these themes. Any differences identified between the states and local areas are noted.

**Table 2:** Summary of emergent themes and exemplar quotes

Theme	Quote	Key Informant Category	Geographic area
Progress being made, overall	It’s more frequent now, believe or not… And the way that I found out was I was out on a call and the guy’s like, ‘Yeah, I gave him some Narcan, but he’s not responding’, like that type of situation. I’m like wow, people are really well aware of this, just like we are	Police officer	Urban
Equity concerns for marginalized communities	It seems like if they had [naloxone] their self or the family members had it, then they would give it before we got to the scene. It seems like we’re seeing more people not have it	EMS provider	Rural
Central role of harm reduction programs in naloxone access expansion	I think our community does a really good job of making [naloxone] available, as far as the needle exchange programs. I think, it’s out there for the ones that do need it	Police officer	Rural
Challenges expanding naloxone access through pharmacies	I have actually, myself, never dispensed it. I have never been working when someone has come in and wanted it	Pharmacist	Suburban
Grappling with stigmatizing attitudes toward people who use drugs and harm reduction approaches	I go back and forth on the whole having Narcan be available for people, because I don’t know if we’re enabling them; like now I know that if I get high, I have this fallback… But then if you don’t have that fallback, are we gonna have more people dying?	Firefighter	Suburban

### Progress being made, overall

There was general agreement among KIs in all three states that more people were obtaining and using naloxone, especially from the perspectives of first responders and harm reduction personnel. Harm reduction personnel described their specific efforts to get naloxone into the community, and many perceived that these efforts had been largely effective.

I think we’re doing a good job of getting naloxone out there. I think one demonstration of this is that we’ve had fewer drive-up overdose situations where somebody drives up, ‘Oh my God, my friend overdosed. I know you guys have naloxone.’ We used to get that very regularly... Now, we don’t see that very often at all. And I tend to think that’s because people are dealing with it themselves or they’re more comfortable calling 911...I mean, we could always do better, but I think we make it pretty accessible, in terms of these efforts, we don’t make it hard to get naloxone.

-Harm reduction personnel, SSP, urban WI

In addition to harm reduction personnel noting progress in getting naloxone out into the community, this KI also suggests that having improved access to naloxone had enabled PWUD to handle overdose events themselves, and potentially increased their willingness to contact first responders. Some first responders suggested that PWUD were more willing to contact EMS during an overdose because they were aware that first responders carried naloxone.

I think the sentiment is users know that if they are experiencing an overdose, there’s a medical intervention that can occur, and if they don’t have Narcan on their person… I think most people realize that emergency medical services has Narcan on board.

-Police officer, suburban CT

Several first responders indicated that naloxone was more often being administered prior to individuals contacting EMS during an overdose. An EMS provider, who worked in a suburban area of CT, noted increases in naloxone being present at the scene of an overdose and attributed these changes to state policies that were geared toward expanding naloxone access through pharmacies. Beginning in 2015, CT state law allowed any pharmacist with the proper training and certification to prescribe and dispense naloxone [23].

KI: We're starting to see Narcan in the house…. In the last year, we're starting to run into more and more cases where people… they used Narcan before we got there or it's sitting on their table and nobody used it or whatever…in the last 12 months, it's way more common than prior.

I: What do you associate with this improvement?

KI: Well, I think the, the legislation didn’t hurt. We passed some legislation making it more accessible 2 years or 2 sessions ago now…It might have been a little bit longer ago…and that seemed to proliferate very quickly. I don’t know the process if it's being prescribed to at-risk populations or people are actually just going into a pharmacy and saying, ‘Hey, I want Narcan.’ I don’t know where they're getting it, but we know that it showed up shortly after the law changed**.**

**-**EMS provider, suburban CT

As the previous passages highlighted, naloxone is becoming more accessible to community members, particularly among PWUD and in the metropolitan areas of CT and WI. Some KIs attributed improvements in naloxone access to policy changes and increased community awareness of opioid overdose prevention.

### Equity concerns for marginalized communities

Despite overall perceptions of progress regarding naloxone access, harm reduction personnel in WI expressed concerns regarding their outreach to marginalized groups, such as individuals in non-English speaking communities and communities of color.

I mean as far as I remember, there’s been plenty of black and brown people that are participating. Exchanging needles and saving their peers with naloxone… But, considering [this] is a majority people of color city, white people are over-represented in this program. So, I don’t know if… it’s just more white people injecting drugs or if there’s a lot of people of color that aren’t engaging in this program.

-Harm reduction personnel, SSP, urban WI

The likelihood of gaps in rural communities was another concern for harm reduction personnel, who indicated that there were less resources available to these communities.

We're kinda more spread out relative to other Connecticut communities. I mean, we're certainly not anything like the frontier communities out in the far west, but parts of… our communities are considered rural, at least here in eastern Connecticut. And so, no, we don't have any needle exchange programs or things, that type of harm reduction, per se… and because of our rural nature… with limited resources and a smaller population to influence, there's only so much we can do.

-Harm reduction personnel, health department, rural CT

As the previous passages highlight, it is likely that there are communities where naloxone is less available, particularly for marginalized groups and in predominantly rural areas, where there may be reduced access to SSPs.

### Central role of harm reduction programs in naloxone access expansion

KIs largely credited local harm reduction organizations, including SSPs and public health departments, for making naloxone more accessible. First responders highlighted how harm reduction programs were effectively getting naloxone out into their communities. In rural KY, an EMS provider spoke of the work by the local health department and other community organizations to improve community access to naloxone.

We are not allowed to distribute anything as far as medication-wise goes, even Narcan, but we have health departments...We have a lot other outreach programs that have classes that are open to the public, and they actually give out Narcan, nasal Narcan...Everyone that attends gets a free sample of it.

**-**EMS provider, rural KY

The work to expand access has become a key focus for many harm reduction organizations and personnel shed light how they were able to get naloxone into the hands of their clientele. They described their capacity to provide naloxone for free and to offer it as part of a menu of many other harm reduction services. In addition, they explained how they make a concerted effort to engage with PWUD, which could include using mobile units or accommodating their clients’ schedules by making services available on the weekend.

So, everything we do is pretty much free. Everything. Free testing, free needles; free Narcan, free fentanyl strips. So, we make it really, really accessible for people...We also have a Saturday site where we just park the van somewhere near here. They kind of do that for people who couldn’t come in during the weekday during business hours. We really try to convey that as well to people… ‘Please come in anytime you need. Please call the van anytime you need.’

-Harm reduction personnel, SSP suburban WI

Community outreach was another area that was described by harm reduction personnel. In addition to them engaging with PWUD, many provided free naloxone and training on how to administer it to their communities at large.

We do a weekly community training – free to anybody who wants to come in. The only thing we ask them is to bring somebody with them. It could be anybody. Because if you need naloxone, you can’t give it to yourself...It varies how many people come, and the interesting thing is that...the majority of the people who have been coming are people...either they’ve had experience themselves with somebody in their family who has overdosed – some of them have unfortunately passed on. And so, you know, they’re coming in to get that kind of information.

-Harm reduction personnel, urban CT

The longstanding mission of harm reduction organizations, especially SSPs, to improve the public health of their communities has been a critical piece to expanding naloxone access to PWUD, as well as to increasing community awareness regarding opioid overdose prevention. The acceptance of a harm reduction philosophy and non-stigmatizing attitudes toward PWUD has facilitated these efforts. Yet, personnel across states and local areas explained that there were still challenges to expanding their capacity to distribute naloxone, particularly inadequate funding.

You know we’re always struggling to have Narcan. We just don’t seem to have enough. So, if there was funding, we could save a lot of lives. We can give it away as freely as we do the needles... ‘cause it’s no good sitting in the warehouse. And we just don’t have the funding to get it.

-Harm reduction personnel, SSP, urban CT

The frustration that harm reduction programs could be doing more if they had better resources was shared by other KIs. Other barriers centered around state and local policies, like requiring physician approval to distribute naloxone.

KI: I think that some of the new funding coming down for overdose prevention sometimes, as great as that is, we have to follow science, which it says putting the Narcan in the hands of users is the most effective way of preventing overdose. And oftentimes the monies tend to go to system responders first, and communities and users last.

I: Are you able to provide naloxone to users?

KI: At this time, we don’t...Our area network is getting a MD [medical doctor] to sign onto what’s required legally to be able to do that, which is called a standing order. Once I get that MD to sign on, we can go live here. That’s what I’m currently working on now.

-Harm reduction personnel, SSP, urban WI

The presence of structural barriers to naloxone expansion were echoed by health departments in KY, where there was generally less progress by harm reduction programs regarding efforts to dispense naloxone in the community relative to the other states. A rural health department official cited limited resources as the primary barrier to expanding naloxone access, and the resulting inequities that can occur in under-resourced areas.

We need to continually advocate for what’s going on but some of our policymakers are so far removed. When you look at public health funding, the bigger health departments, where the services are… the best-funded health departments now also have the best health outcomes.

-Harm reduction personnel, health department, rural KY

In fact, at the time of the interviews (2019), some areas in KY were still in the process of getting naloxone out to their community members. Beginning in 2015, the KY Department of Health provided guidelines for local health departments to establish harm reduction programs, but approvals are required at multiple levels, including the local health department and both the city and county government.

### Challenges expanding naloxone access through pharmacies

Gaps in naloxone expansion efforts were also highlighted by pharmacists. Overall, pharmacists did not indicate a demonstrable shift in their dispensing of naloxone within their communities, despite there being a standing order in WI and KY, and CT pharmacists having prescriptive authority. However, one pharmacist suggested that clients were more likely to obtain naloxone at the pharmacy if it was prescribed by their physician.

I have had more of our chronic pain management doctors to say you should really have this on hand just in case. And I will say that the majority of patients, when they tell them that their prescribers want them to have it on hand, will take it.

**-**Pharmacist, rural WI

Another pharmacist suggested that, even when they initiated conversations around naloxone in the pharmacy, many of their clients did not see a need for it and could be further dissuaded from obtaining it due to cost.

I try to talk to patients, like any opioid prescription can overdose. What to look for, the signs and symptoms of overdose. And I offer naloxone, but most people don’t care. I’m not gonna abuse the medication. Like they take it personally for some reason...I try to explain to them, it’s not about you abusing medication. You might forget, especially if it’s an older person, you might forget and you take an extra tablet… some people actually will agree, others… don’t want it—especially if the insurance is not covering.

**-**Pharmacist, urban WI

In addition to the out-of-pocket cost of naloxone being a barrier, the pharmacist in the previous passage notes that some individuals are offended if naloxone is prescribed or brought up in the clinical encounter. The preceding passage also indicates that when consumers are obtaining naloxone in the pharmacy, insurance coverage can play a significant role in determining affordability. A consumer is put in a situation where they must weigh the out-of-pocket expenses with their perceptions of the medication’s overall value to their personal situation. In situations where the perceived level of risk is low, there may be less willingness to pay for the medication—assuming an individual has the money to spend. In situations where the risk is perceived as high, consumers may still be in the unfortunate position of simply being unable to pay. Harm reduction personnel also noted affordability as a barrier that their clients faced in accessing naloxone from pharmacies.

If you’re not coming in to one of our offices and getting it for free, you’re paying for it over the counter, which can be hundreds of dollars for one or two doses...[and] the average person doesn’t have a couple hundred dollars to spend on something just in case.

-Harm reduction personnel, SSP, rural WI

Translating expanded naloxone access policies into practice was an additional challenge brought up by pharmacists. In CT, although pharmacists have prescriptive authority to dispense naloxone, they must be certified through the state to do so. A pharmacist, who worked in an urban area of CT, described how pharmacies may not necessarily have a pharmacist onsite who is certified to dispense naloxone.

The ability of the community pharmacist to dispense naloxone nasal spray without a prescription, I think that has been, that law is really good. In practice, it doesn’t always work well...It was maybe a year ago, I went to all the pharmacies like around the… area like [large retail pharmacies] and so on, and I said, you know, can I have a prescription for Narcan nasal spray. Oh, no. The pharmacist here doesn’t have a provider number, so they can't give it to you… And, actually, the night I was looking, there was nobody around. So, that is not good.

**-**Pharmacist, urban CT

Although pharmacists have a critical role to play in expanding access to naloxone, the prior passages indicate that it takes more than changes in policy for this to be realized. Strategies that can address the attitudes and awareness of pharmacists and consumers are also necessary, as well as improving the affordability of naloxone when obtained in the pharmacy.

### Grappling with stigmatizing attitudes toward PWUD and harm reduction approaches

While there was general consensus among KIs that getting naloxone into the hands of individuals at high-risk for overdose was important and effective in reducing opioid-related overdose deaths, many KIs still struggled with this as a public health intervention. Several first responders described the tension they felt around expanded naloxone access, including the perception that it enabled riskier drug use behaviors. This tension, around the acceptability of naloxone, was well-reflected in a statement by a pharmacist in KY, who expressed tension with respect to dispensing naloxone to PWUD, as well as ambivalence toward other harm reduction services, like syringe exchange.

I have a lot of mixed emotions about let’s prescribe something, so someone can go out and party hardy. Then they have an overdose and it kind of brings them back. I have some issues about that. It’s like I have issues about the needle swap.

**-**Pharmacist, rural KY

Hesitancy, on the part of pharmacists, to endorse a harm reduction approach to opioid overdose prevention was not lost on harm reduction personnel. One related the current environment, of obtaining naloxone through the pharmacy without a prescription, with access to sterile syringes from the pharmacy during the HIV/AIDS epidemic. Although previous legislation in the 1990s allowed CT pharmacies to legally sell sterile syringes, pharmacists could still exercise discretion in making them available to local consumers, which could limit access—particularly in non-urban areas or where negative attitudes of PWUD were more pervasive [45].

You know, you can’t always get naloxone at a pharmacy...depending on what pharmacy you go to and what you look like when you walk in the door…. If you have medical insurance, you can go to your pharmacy and the pharmacist will give you a five-minute little lecture on how to use it. And he or she will charge it off on your insurance, you know? I know [someone] who went through the whole thing herself. She shared that with us one day…what a hard time it was for her to get it…It’s kind of like when Connecticut changed the laws around hypodermic needles, you know. Once upon a time, you needed to have a prescription to get hypodermic needles. And then back during the AIDS epidemic, Connecticut was one of the few states that changed regulation so that people could buy’ m in the pharmacy without a prescription**.**

-Harm reduction personnel, SSP, urban CT

Concerns that PWUD may encounter stigma at the pharmacy when trying to obtain naloxone were validated by the comments of a pharmacist in suburban WI, who had observed stigmatizing attitudes toward PWUD and related it to a lack of awareness that many healthcare providers have regarding addiction and the opioid epidemic.

I think the pharmacists who I see who are just like leave, get out of my store, I don’t wanna deal with you...I think they need to be educated on just the history of how people become addicted. It started out as a legitimate pain issue and they were given something that gave them that first high and now they keep seeking it, and just make them understand—having maybe a little more empathy. I see that as missing as far healthcare providers**.**

**-**Pharmacist, suburban WI

A pharmacist in KY suggested that asking everyone who obtained opioid medications if they had access to naloxone was a potential strategy to increase uptake and to reduce bias and stigma around naloxone distribution in the pharmacy.

Rather than pick and choosing, because you don't want to necessarily make somebody think that you're singling them out for whatever reason... some pharmacies will ask every opioid prescription, ‘Do you have naloxone at home? Do you know what naloxone is? Do you understand why it might be?’... and then you have to tell people, ‘I'm not saying you're a user. I'm not saying you're an addict...but you still could be at risk for overdose just by virtue of having this in your house. Can I talk to you about it?’ So, it kind of varies but studies have definitely shown that when it's mandated, when it's required to prescribe it at the same time, the dispensing of it goes up as well.

**-**Pharmacist, urban KY

Notwithstanding the negative attitudes that some first responders and pharmacists had toward expanded naloxone access, others suggested that the opioid epidemic had influenced a cultural shift toward a harm reduction approach in their sectors. One pharmacist detailed an initiative in KY to get pharmacists into the community using an existing mobile pharmacy unit.

When I came into this role, I actually was doing emergency preparedness because the state owns a mobile pharmacy surge unit. It's an emergency preparedness asset… and the goal of it is just to be a mobile pharmacy in times of disaster. [In] 2015, Senate Bill 192, the heroin bill, was passed and that for the first time allowed pharmacists to distribute naloxone via physician protocol. So, it basically made it to where you didn't have to have a prescription from your doctor... The next year, 2016, there was some discussion about, ‘Hey, we've got this mobile pharmacy...Have we thought about getting in community and doing naloxone education events with pharmacists? To utilize it in the protocol?’ It was very successful... And so that started growing and more money started to flow into the state from the federal level for the opioid crisis...We were able to spin-off and have a dedicated pharmacist just for naloxone aspects… we were able to procure a... mobile harm reduction unit...and the whole purpose of it was to be able to dispense naloxone, potentially do vaccinations or harm reduction sort of activities that would need a pharmacist.

**-**Pharmacist, urban KY

A firefighter in WI described a cultural shift in the local overdose prevention response, and the fire department’s ability to leverage an existing community paramedics program. The program evolved to embrace a more holistic approach to addressing the needs of PWUD, including the adoption of a harm reduction philosophy.

What we [the fire department] have done… is we have chosen to utilize our community paramedic program that already has some really good established partnerships, both public and private, to create a system where we will identify the overdose patient who has received Narcan… our community paramedic team will be activated immediately, and then resources will be pooled around this person to try to get them as much help as possible… Focus on giving them Narcan… focus on any housing needs they may have, any behavioral health needs they might need, all sorts of different socioeconomic areas where we can surround them with and get them the help they need.

**-**Firefighter, urban WI

The programs highlighted in the previous passages shed light on how some pharmacists and first responders, especially in urban areas, are adapting harm reduction approaches to respond to the opioid crisis in their communities. Yet, it remains clear that stigma toward PWUD and harm reduction is an ongoing challenge among these sectors, and it is likely these negative attitudes are pervasive within their communities.

## Discussion

The opioid epidemic has claimed nearly a half a million lives to date in the U.S., making overdose prevention a national priority [46]. However, many prevention efforts, such as increasing naloxone access, are implemented largely at the state and local level. This study sheds light on the shifting landscape of naloxone access across communities in CT, KY, and WI, and the perspectives of KIs on the local context of naloxone access within these communities. While there was general agreement across states that naloxone access had improved, overall, there were concerns regarding inequities in access for marginalized and under-resourced communities—especially rural areas. Study findings indicate that efforts to support expanded naloxone access through harm reduction programs have been particularly effective, and less so in pharmacies. Additionally, findings underscore how negative attitudes toward a harm reduction approach and PWUD represent a critical threat to existing and future efforts to expand access to naloxone.

Study results indicate that harm reduction programs were particularly effective in increasing access to naloxone at the local level, especially for individuals who are high-risk, such as PWID. These findings are consistent with previous research that has found PWUD are more likely to have obtained naloxone from harm reduction programs [10, 11]. When adequately resourced, harm reduction programs are well-positioned to increase naloxone access in their local areas. They can develop trusting relationships with individuals who are at high-risk for overdose and their philosophy to ‘meet people where they are’ is often literal, by providing services in a mobile unit and for free, as well as being available at night and on weekends [47]. Further, harm reduction programs often take on additional overdose prevention strategies, such as community outreach events that can increase awareness among individuals who would not typically seek out their services [9]. Yet, our findings indicate that harm reduction programs can face structural challenges to expanding naloxone access, especially in areas where there are fewer resources dedicated to harm reduction. The barriers highlighted by KIs provide context regarding why gaps in naloxone access can exist at the local level.

Study findings underscore significant challenges to accessing naloxone at the pharmacy, despite laws that have passed in each state to provide pharmacists with a standing order (KY, WI) and prescriptive authority (CT) to distribute naloxone. In addition to out-of-pocket expenses being a barrier, due to a lack of insurance coverage or plans not fully covering naloxone, there were barriers regarding consumer awareness and negative perceptions of naloxone by some consumers and pharmacists. Limited naloxone access in the pharmacy environment is problematic for a few reasons. Although harm reduction organizations may be a better option for many, the current study and previous research has shown that they are not available to every community [15]. PWUD, but do not inject, may also be disengaged from or uncomfortable going to SSPs to obtain naloxone. Pharmacy-based naloxone interventions have been shown to be a feasible and acceptable approach to overdose prevention and there are consumers who may prefer accessing naloxone from a pharmacist for various reasons, such as an existing relationship or a preference for receiving health information from a health care provider [33, 48]. To mitigate barriers to naloxone access in the pharmacy environment, initiatives that encourage universal screening have the potential to increase awareness among consumers and reduce negative perceptions held by both pharmacists and consumers [49]. Full coverage by insurance companies could also help reduce financial barriers and would be consistent with how many other preventive health services are covered [50].

Findings indicate that stigmatizing attitudes towards harm reduction approaches and PWUD remain a key challenge to expanding naloxone access [17, 51]. These findings are consistent with prior research from the perspective of PWID who had accessed pharmacy services for harm reduction [52]. There were perceptions among first responders and pharmacists, in particular, that having access to naloxone could enable risker drug use behaviors, or motivate moral hazard, because PWUD have a “fallback” to reverse an opioid-involved overdose. The moral hazard concern is certainly valid [53] and elucidated a point of tension among KIs, who also acknowledged that the alternative—not providing lifesaving medication—was an even less desirable option. These tensions, likely exacerbated by being on the frontlines of a seemingly intractable drug epidemic, have practical implications regarding stigmatizing attitudes toward PWUD and overdose prevention strategies and may extend into the broader community. High levels of stigma within a community can make it more challenging to garner support for harm reduction programs, and may discourage high-risk individuals from seeking out overdose prevention or substance use treatment services [17, 54]. Notably, this study also highlighted local efforts, by a pharmacist and first responder, to play a larger role in overdose prevention and to be more accepting of a harm reduction approach. An evaluation of these types of local initiatives and their outcomes would be useful in determining their effectiveness in preventing overdoses and other opioid-related harms, and may pave the way for greater community support and funding for harm reduction programs.

The current study did have some limitations. The study’s participants were a subset of KIs who were recruited for Project LEAD. There is geographic variation within the KI sample and differences in how study sites identified participants within their respective states. Further, while the KI interview guides explored specific questions related to naloxone administration and distribution, the KI sample was not recruited to directly address the current study’s aims [55]. The study data are also based on information that was self-reported, which has the potential for recall bias, and the nature of the data do not allow for causal relationships to be drawn [56]. Finally, the study’s findings are from the perspectives of individuals in the community who dispense and administer naloxone, versus individuals who are seeking to obtain naloxone within the represented communities. The lived experiences of consumers, who are trying to access naloxone in their local communities, is an important area of emerging research [57–59], and it is critical to develop a better understanding of how those experiences may differ by state and local area. Notwithstanding these limitations, the current study provides novel insight into the social context of naloxone access, and to the authors’ knowledge, is the first to highlight how differences in the local context can influence the implementation of naloxone expansion strategies.

## Conclusion

Expanded access to naloxone is an important public health strategy to reduce opioid-involved overdoses. Study findings indicate progress in each of the three states studied, but identified gaps in naloxone access, especially in rural areas that have fewer harm reduction programs dispensing naloxone. In addition, our findings suggest that policies to expand access through the pharmacy have been less effective than providing resources to harm reduction organizations that have longer established relationships with PWUD who are at higher risk for overdose. Multilevel strategies to reduce stigma toward PWUD and harm reduction approaches appear warranted within efforts to expand community access to naloxone, particularly among pharmacists and first responders. Enhanced resources to develop and expand harm reduction programs in local communities represent another key opportunity, as well as insurance companies providing full coverage for naloxone as they do for many other preventive services.

## Supplementary Information

**Additional file 1.** Key informant interview guides for first responders, harm reduction personnel, and pharmacists.

## Data Availability

Supporting data is not available due to ethical restrictions in place to protect the privacy of research participants, who did not provide consent for their data to be shared publicly. Inquiries related to supporting data availability should be directed to the principal investigator of the study (JDG) at jdickson@mcw.edu.

## Abbreviations

CT: Connecticut
EMS: Emergency medical services
KIs: Key informants
KY: Kentucky
PWID: People who inject drugs
PWUD: People who use drugs
SSP: Syringe services program
U.S.: United States
WI: Wisconsin
