# Supplementary material for: A qualitative examination of naloxone access in three states: Connecticut, Kentucky, and Wisconsin
Source: BMC Public Health. 2022 Jul 19;22:1387. doi: 10.1186/s12889-022-13741-5 (PMC9295344; doi:10.1186/s12889-022-13741-5)
Supplement: Supplementary file 1 — Additional file 1. Key informant interview guides for first responders, harm reduction personnel, and pharmacists. [file 12889_2022_13741_MOESM1_ESM.docx]

**Additional File 1. Key informant interview guides for first responders, harm reduction personnel, and pharmacists**

***First Responders***

1. How often do you see opioid overdoses? How are you able to respond to these?
2. Who reports the emergency? Have you seen changes in drug users’ willingness to call first responders in cases of overdose?
   1. To what do you attribute change or lack of change?
   2. How aware do you think PWUD are of Good Samaritan laws? How often do PWUD report overdoses?
   3. How many PWUD do you think carry naloxone? How often do you think a person who uses opioids actually has naloxone available when they need it for themselves or an associate?
   4. What would need to be done to improve this?
3. Have you seen changes in the number of deaths due to overdose? To what do you attribute any change or lack of change?
4. How often do you see acute intoxication with other substances? How often do you see drug mixing? How do you respond to these?
   1. How willing are persons who are experiencing a drug crisis to tell you what they have taken?
   2. Do PWUD in acute distress have legal immunity for use in your state? What is the impact of this, if any?
5. Do you have other facilities to take PWUD who may be acutely intoxicated or in a mental health crisis other than the police station?
   1. What are some of these places? How well do they work in addressing the problems of PWUD?
   2. If no such places exist, what are some barriers to alternative spaces?
   3. How has injection drug use changed in the last five or ten years? Who injects? When do they start injecting?
6. What are the most popular drugs on the street? What are the most popular opioids on the street? (Brand and street names.)
   1. Does your work give you a sense of changes in the availability of different types of drugs on the street? What changes have you seen and around when did you see changes?
7. Have you seen changes in the proportion of injection versus non-injection opioid users over time (oral or intra-nasally)? What kinds of changes have you seen? What do you think has caused some of these changes?
8. Have you seen changes in the availability of prescription opioids on the street?
   1. What about heroin?
   2. If so, when did you see the change?
   3. What do you attribute the change to, like certain laws?
9. Do you always have access to naloxone? If no, what are some of the barriers to getting naloxone?
10. Do you hold trainings and/or distribute naloxone? What have you liked and disliked about these trainings?
11. What kind of trainings have you received to increase your own safety when dealing with opioid using or injection drug users? Needle stick injuries? Gloves and safe handling for Fentanyl?
12. What other kinds of medical problems have you seen among PWUD that you encounter? Abscesses, endocarditis, etc.?
13. Is there anything we haven’t covered that you believe is important?

*Additional questions for police only (first responder questions still apply):*

1. How and when do you arrest people for possession of opioids? What other options do you currently have and what would you rather see?
2. How do you feel about Syringe Service Programs?
3. What do you think police priorities should be in terms of enforcing current drug laws?
   1. What are current efforts to reduce opioids available for diversion? How effective do you feel these are? Have you seen inadvertent negative effects of these?

***Harm Reduction Personnel***

1. Can you tell me a little about your position and your role? How long have you been working in SSPs and harm reduction and why did you get involved?
2. Where in the state are SSPs or clean syringes easily available and where are they not?
3. What are some challenges in providing SSPs in different areas of the state?
4. What are some of the local challenges to providing SSP services?
5. What have been the reactions of the community to SSP?
6. What other services do you provide?
7. What barriers have impeded your efforts at providing all the harm reduction services you would like?
8. Have there been events or policies that have made it easier to provide services? Any policies or events that have made it more difficult?
9. What is your relationship with law enforcement? What is your relationship with emergency department medical providers?
10. How often do you see secondary exchanges in which PWID provide syringes to associates? Do you see these more often in rural versus urban areas? What do you think are advantages and potential disadvantages to this practice?
11. Have you seen any changes in the population that receives your services? What kinds of changes have you seen? For example, age, knowledge of safe injection practices, kinds of drugs injecting?
12. How have you responded to increase education around safe injection? What kinds of interventions do you provide?
13. Are you able to provide naloxone? What kinds of overdose education do you provide? How effective do you think current effort to reduce opioid overdose are, and what more should be done?
14. Have you seen any changes in the drug of choice (especially opioids) over time? To what do you attribute these changes?
15. Have you seen changes in the availability of prescription opioids on the street? Heroin? To what do you attribute these changes?
16. Is there anything we haven’t covered that you believe is important?

***Pharmacists***

1. Please tell me a little about yourself. How long have you been a pharmacist? What are some of the different places you have worked? What is your role in this pharmacy? Are you a manager or a supervisor?
2. What restrictions are in place to limit opioid misuse or diversion?
   1. What are the sources of these restrictions? Insurance? Prescription drug monitoring programs (PDMPs)? Corporate policy for corporate pharmacies? Pharmacy owners’ policies?
   2. Which of these do you think are working to limit the availability of prescription opioids on the street?
   3. Which of these do you think are not working?
   4. What would you do instead of the current policies?
3. How have policies and laws changed over time? How has that affected your practice?
4. [If pharmacists have worked in different places], how do pharmacies differ in their efforts to control opioid prescription?
5. For opioids prescriptions, do you accept electronic or paper scripts?
6. What are your experiences working with different insurance companies and Medicaid, specifically regarding prescription opioids? What is the burden in complying with restrictions and getting reimbursed?
   1. What are the reasons some opioids are on preferred drug lists and others require prior approval? Abuse potential? Price?
7. Under what circumstances do you accept self-pay for prescription opioids?
8. What are your experiences using the PDMP? What are some of the reasons you check it? What are you looking for? What kinds of things would raise suspicions regarding a physician’s prescribing practices or that a patient may be drug seeking? What do you generally do in these cases?
   1. How much of a burden is it to check the PDMP?
9. Do you believe anyone is monitoring whether you are checking the PDMP? Who is monitoring (headquarters, state PDMP)? How are they doing so? What are the consequences if they find you are not checking the PDMP if required?
10. Do you ever use electronic medical records to check multiple prescriptions, multiple physicians or use of different pharmacies? Is this in addition to, or instead of the PDMP?
11. Have you had many experiences with patients who you think may be misusing prescription opioids? What kinds of behaviors would lead you to believe that someone is drug seeking and not in physical pain? What do you do in these cases?
12. Have you had any experiences in which you suspected that a person was filling a prescription for someone else? What do you do in these cases?
13. Have you had experiences where you suspect that some opioids you dispense are diverted? What do you do in these cases?
14. How much discretion do you have in filling prescriptions? How much discretion do you have in following policies at the pharmacy, insurance or PDMP level?
15. How do PWUD get around the current policies?
16. Do you provide naloxone?
    1. If so, what kinds of efforts have you seen to educate drug users and their loved ones about naloxone and provide naloxone to them? What would make these efforts more effective?
    2. If not, what are some of the reasons you are not providing naloxone?
17. Do you suspect some pharmacists dispense opioids they know will be misused or diverted? How are they managing to get around policies or laws designed to stop opioid diversion or misuse? What kinds of laws/policies are needed to prevent this?
18. Do you distribute syringes without a prescription?
    1. If so, how often do people without a prescription come to buy syringes? What encourages or discourages drug users from buying syringes?
    2. If no, what are some of the reasons you do not distribute or sell syringes without a prescription?
19. What kind of training have you receive regarding the laws and policies that are in place to prevent opioid abuse? What did you like and dislike about the trainings? Are there any areas in which you feel you need more training? What are these?
20. How confident are you that you are complying with the law?
21. Are you concerned about penalties? How so?
22. Is there anything we haven’t covered that you believe is important?
